# Milk Sickness

**Published:** 1879-11

**Authors:** 


### MILK SICKNESS,

Called, by some, *the Trembles*, is a disease prevalent in some parts of the West and South-West, and many have died with it. It is caused by drinking the milk of a diseased cow; a more fatal form of it arises from the use of butter or cheese, made from such milk, or from eating the flesh of any animal fattened with the milk, while the cow herself, nor the animal fattened with her milk, does not necessarily manifest symptoms of disease. One of the first questions of many seeking homes in the far west is, Has milk sickness ever been known here? Some of the finest lands in the world are without a market because the disease is in the neighborhood.

Soon after swallowing the milk, the person has thirst, nausea, swimming in the head, vomiting, fever, skin hot, eyeballs blood-shot, excessive debility, paralysis, oppression, stupor, hiccup, and death. In some cases, the heart beats with such violence as to strike the by-standers with horror, and even alarms the physician who has never witnessed it before.

*Well fed cows never give milk-sickness.* I have revelled in the use of the most luscious milk, and the most delightful fresh butter for weeks together, in perfect fearlessness of milk-sickness, when several persons had just died of it on the next farm. The reason was, the cows were fed night and morning in winter with as much corn and meal as they wanted, and had sweet hay to eat during the day, and plenty of it; while, in the summer, they had fresh pasture and still something to eat of the slops of the kitchen at milking times, and knowing they would get something good, they never failed to come of their own accord, they thus literally "rolled in fat," summer and winter.

Some persons have attributed it to one vegetable, or weed, or grass; others to drinking from a certain spring, each locality having a different plant; these differences of opinion, together with the conceded fact, that it is not known on a well cultivated farm, are proofs, in my mind, of the truthfulness of the opinion which I have suggested.

